# Differentiation between Diphtheria and Membranous Croup

**Published:** 1886-11-20

**Authors:** W. A. Morris

**Affiliations:** Austin, Texas


### DIFFERENTIATION BETWEEN DIPHTHERIA AND MEMBRANOUS CROUP.

BY DR. W. A. MORRIS, M D., AUSTIN, TEXAS.

Having carefully read the discussion by the gentlemen constituting the Galveston Medical Club regarding the differentiation between diphtheria and membranous croup, I must say, that I fully indorse the views advocating the duality of these diseases as expressed therein.

I have given much time and thought upon the subject and have also had considerable experience in the treatment of both maladies; and from my standpoint of observation I cannot see how it is possible for a practitioner of any experience to look upon them as in any way allied affections. In the discussion, many of the most prominent points of difference were brought out in bold relief. I will, however, present a few more of a marked character that are never found in common to both diseases.

Before this country was visited by diphtheria, we never found paralysis following croup; and while croup prevailed from year to year no diphtheria occurred, or diphtheritic complications. Paralysis may be partial—affecting the vocal cords with partial loss of voice; may affect the eyes, one half the body, the heart and sexual organs.

I treated a case in 1861. The paralysis was general, completely involving every muscle of the body. It followed a mild case of diphtheria two weeks after convalescence had set in. At first I noticed a staggering gait like that of a drunken man; his head dropping from side to side. He would frequently fall. He soon became unable to walk at all, and lost all power to control his head or any of the voluntary muscles. The power of deglutition partially destroyed and the heart's action feeble and tumultuous. He could be rolled about like a log; death seemed inevitable. But after remaining in that condition one or two days an improvement was visible and recovery finally ensued. Again, some attacks of diphtheria may be ushered in with such severity that the shock to the sympathetic nervous system from blood-poisoning produces death in a few days, even without laryngral complications, or it may take place at a later period from septicemia. In such cases I have seen the pulse drop to 48 or 50 per minute, with persistent vomiting, palor and great prostration; and the rule is, death in a short while.

Again, in the later stages of this disease, when decomposition of the pseudo-membrane begins, there is a constant drainage from the mouth of a dark sanious character, containing shreds or flakes of decomposed membrane of an extremely offensive odor, sickening to the attendants. It is sometimes so free that it becomes necessary to keep the little patient on the side in order to prevent the fluid passing into the stomach, thereby further poisoning the system or pouring into the glottis, producing death by apnœa.

I confess that I see in diphtheria an infectious and contagious disease, zymotic, idiopathic, distinct from any other disease, having an identitity of its own; and when there are complications, as are often found in croup, scarlet fever, and in many other affections, it is only when diphtheria is prevailing, either as an epidemic or in its sporadic form. I will here tabulate the differentiation embracing the whole subject.

| DIPHTHERIA. | CROUP. |
|---|---|
| 1. Asthenic................ | 1. Sthenic.................. |
| 2. Tonsils, pharynx and soft palate first involved.......... | 2. Larynx and trachea first involved..................... |
| 3. Dribbling from the mouth fœted sanious fluid mixed with shreds of decomposed membrane. | 3. Mouth and throat usually dry.......................... |
| 4. Epidemic................ | 4. Never epidemic......... |
| 5. Constitutional with blood changes..................... | 5. Local.................... |
| 6. Lymphatics of neck greatly involved. | 6. Slightly if at all......... |
| 7. The pseudo-membrane involves the mucous and submucous tissue................ | 7. Upon the mucous tissue. |
| 8. Infectious and contagious. | 8. Neither.................. |
| 9. Hemorrhagic ............ | 9. Not hemorrhagic........ |
| 10. The deposit forms upon blistered and other abraded surfaces and wounds......... | 10. Not so in croup......... |
| 11. Paralysis follows mild as well as severe cases, involving motor muscles of eye, pharynx, larynx, sexual organs, etc..... | 11. No paralysis at all....... |
| 12. Pathological changes are found in the brain, heart, kidneys and other organs........ | 12. In these organs no pathological changes............. |
| 13. Death results sometimes from paralysis, toxæmia, septicæmia...................... | 13. Never.................. |
| 14. Albuminuria............. | 14. No Albuminuria. |

—*Texas Medical Journal.*

